# Transcriptional Analysis on Resistant and Susceptible Kiwifruit Genotypes Activating Different Plant-Immunity Processes against *Pseudomonas syringae* pv. *actinidiae*

**DOI:** 10.3390/ijms23147643

**Published:** 2022-07-11

**Authors:** Xiaobo Qin, Min Zhang, Qiaohong Li, Dalei Chen, Leiming Sun, Xiujuan Qi, Ke Cao, Jinbao Fang

**Affiliations:** 1 Zhengzhou Fruit Research Institute, Chinese Academy of Agricultural Sciences, Zhengzhou 450009, China; qin_ever@hotmail.com (X.Q.); zhangmin1862@163.com (M.Z.); xqrenjia@126.com (Q.L.); chendalei@caas.cn (D.C.); caoke@caas.cn (K.C.); fangjinbao@caas.cn (J.F.); 2 Sichuan Provincial Academy of Natural Resource Sciences, Chengdu 610015, China; 3 Key Laboratory of Coarse Cereal Processing, Ministry of Agriculture and Rural Affairs, Chengdu University, Chengdu 610047, China

**Keywords:** kiwifruit, transcription, resistant, susceptible, *Pseudomonas syringae* pv. *actinidiae*

## Abstract

*Pseudomonas syringae* pv. *actinidiae* (Psa), a bacterial pathogen, is a severe threat to kiwifruit production. To elucidate the species-specific interaction between Psa and kiwifruit, transcriptomic-profiles analyses were conducted, under Psa-infected treatment and mock-inoculated control, on shoots of resistant Maohua (MH) and susceptible Hongyang (HY) kiwifruit varieties. The plant hormone-signal transduction and plant–pathogen interaction were significantly enriched in HY compared with MH. However, the starch and sucrose metabolism, antigen processing and presentation, phagosome, and galactose metabolism were significantly enriched in MH compared with HY. Interestingly, the MAP2 in the pathogen/microbe-associated molecular patterns (PAMPs)-triggered immunity (PTI) was significantly up-regulated in MH. The genes RAR1, SUGT1, and HSP90A in the effector-triggered immunity (ETI), and the NPR1 and TGA genes involved in the salicylic acid signaling pathway as regulatory roles of ETI, were significantly up-regulated in HY. Other important genes, such as the CCRs involved in phenylpropanoid biosynthesis, were highly expressed in MH, but some genes in the Ca^2+^ internal flow or involved in the reactive oxygen metabolism were obviously expressed in HY. These results suggested that the PTI and cell walls involved in defense mechanisms were significant in MH against Psa infection, while the ETI was notable in HY against Psa infection. This study will help to understand kiwifruit bacterial canker disease and provide important theoretical support in kiwifruit breeding.

## 1. Introduction

Kiwifruit, belonging to *Actinidia*, is an important economic fruit crop, mainly produced in China, New Zealand, Italy, and so on. The bacterial canker of kiwifruit is a kind of economic bacterial disease caused by the bacterial pathogen *Pseudomonas syringae* pv. *actinidiae* (Psa) and is a major constraint to kiwifruit production. This disease was first reported on *Actinidiaechinesis* var. *deliciosa* in Shizuoka, Japan in 1984 [1]. Psa can degrade lignins and phenols, which are conducive to its growth [2], and can effectively absorb iron ions from the plants to enhance its virulence [3,4]. Until 2010, the pathogen of Psa was detected in New Zealand, and, so far, it has been detected in the main kiwifruit-producing countries, such as China, Chile, and European countries [5,6]. The disease’s incidence and infection patterns are consistent throughout the world. The cultivars of kiwifruit are generally sensitive to Psa, such as the current master cultivars in New Zealand and China, “Hort 16A” and “Hongyang”, respectively. As an epiphytic bacterium, the pathogen of Psa can be colonized on the surface of the plant tissues, including flowers/pollen, leaves, and stems [7]. Psa enters the vine through openings such as natural stomata and wounds. Then, it can infect the flowers/pollen, leaves, and stems, successively colonizing and occluding the xylem vessels and phloem-sieve tubes for systemic infection [8,9], which causes symptoms including dark brown spots surrounded by yellow halos on leaves, shoot dieback, the wilting of buds, and the oozing of opalescent or red rusty exudates on canes and trunks [10,11]. The infected male flowers produce contaminated pollen, which could transmit Psa to healthy plants [7]. Low temperatures (12 °C–18 °C) are particularly conducive to the propagation and rapid spread of Psa. Spring and autumn are the most favorable seasons for Psa infection [12]. Kiwifruits are infected with Psa on a large scale in a short time, which is inseparable from its infection principles. Thus, it is important to study the mechanism of resistance.

For the resistance mechanism, previous studies are clear, mainly including that it is well known that plant growth is often influenced by abiotic and biotic stresses. Plants have evolved to form a series of complex adaptation mechanisms against pathogens. These defenses included pathogen-associated molecular patterns (PAMPs)-triggered immunity (PTI), and specific bacterial-effector-triggered immunity (ETI) [13,14]. PTI recognizes molecular microbial determinants through pattern recognition receptors, which activate a mitogen-activated protein kinase cascade that results in defense responses [15]. Pathogens can evolve a series of effectors to inhibit the recognition of host cells; but these effectors will interact with resistance proteins to elicit ETI. ETI can cause systemic plant-acquired resistance and activate the plant’s hypersensitivity response, which even follows a massive burst of reactive-oxygen species (ROS) at pathogen-infection sites. Meanwhile, many natural sugars play a role in ROS production [16]. Natural metabolites such as salicylic acid (SA) often act as regulatory components of a plant’s innate immunity processes. SA is a key regulator of a plant’s basal immunity to biotrophic and hemibiotrophic pathogens [17,18,19].

Studies on other pathovars of *P. syringae* give us the chance to view the interactivity between *P. syringae* and the host. The genome of Psa has been analyzed by different groups, and the genes possibly involved in pathogenesis were identified [20]. For kiwifruit bacterial canker, the transcriptomic profile of kiwifruit infected by Psa was reported, but it is from the analysis of a single cultivar [21]. We performed transcriptome-sequencing analysis of the resistant kiwifruit cultivar Maohua (MH) and the susceptible cultivar Hongyang (HY) after infection with Psa. Furthermore, differentially expressed genes (DEGs) were analyzed after Psa inoculation, and some active factors causing kiwifruit bacterial canker were discussed. This study will help to explore the biological pathways and resistance genes involved in kiwifruit bacterial canker disease and to lay the foundation for understanding the pathogenesis of kiwifruit plants against Psa.

## 2. Results

### 2.1. Symptoms of MH and HY Shoots Infected with Psa

**Figure 1:**
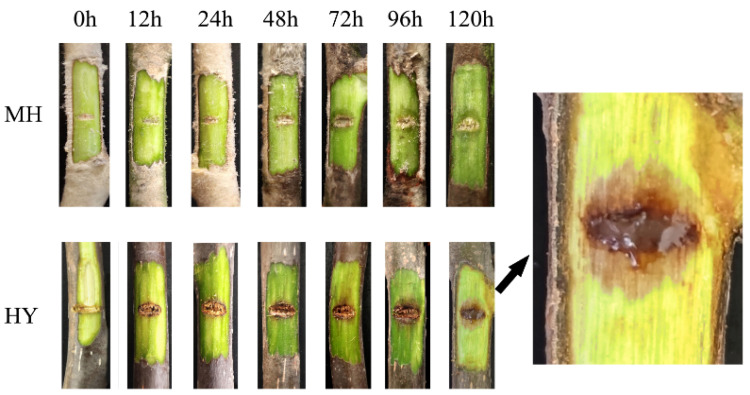
The phenotypic changes of disease symptoms in *Actinidia* MH and HY at 0, 12, 24, 48, 72, 96, and 120 hai (hours after inoculation) with Psa.

There are obvious differences in resistance to Psa between the infected kiwifruit varieties MH and HY. So, MH and HY kiwifruits were selected to observe phenotypic changes after Psa inoculation (Figure 1). Both mock-inoculated genotypes used in this study showed no symptoms of infection. After exposure to Psa, the HY shoots showed clear symptoms. For example, the inoculation area turned brown, and the lesion length was about 1.08 mm at 12 h. After that the lesion length gradually increased between 24 h and 144 h. In contrast, no brown appeared on the inoculation area of the MH shoots. However, the lesion of the MH shoots looked like a water spot, which was observed at 72 h. Notably, another typical symptom exhibited, milky-white bacterial pus, was just observed in the HY shoots 120 h after Psa infection. In addition, the symptom was not observed in the MH shoots. The results reveal that the MH variety is resistant to Psa infection, while the HY variety is susceptible.

### 2.2. RNA-Seq Data Analysis and DEGs in Response to Psa Infection

**Figure 2:**
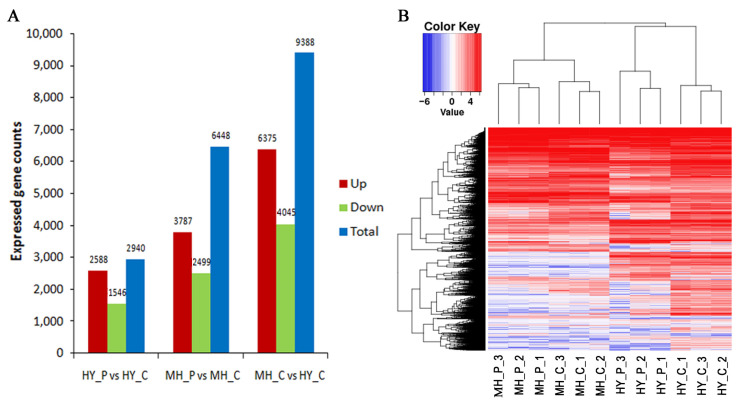
The number of the DEGs identified (**A**) and heat map (**B**) for MH and HY with Psa.

Based on the shoots’ infection results, RNA-Seq analyses were performed on HY and MH at 72 h after inoculation (hai). The samples were made with three biological replicates at each time point. The raw reads varied from 47.13 to 53.84 million. After filtering and trimming, the clean reads varied from 41.93 to 47.80 million. Uniquely mapped reads accounted for 35.15% to 87.93% of the total mapped reads on the Kiwifruit Genome Database (Table 1). According to the total mapped reads, the expression levels of 34,531 genes were calculated using the FPKM method. The number of DEGs between HY and MH was examined at 72 hai with Psa (genes with *p* < 0.05 found by DESeq were assigned as differentially expressed. Comparing with an HY control, the numbers of up-regulated genes and down-regulated genes in HY_P (HY inoculated at 72 hai) were 2588 and 3787, respectively (Table S1); comparing with an MH control, the numbers of MH _P (MH inoculated at 72 hai) were 1546 and 2499, respectively (Table S2). Overall, the number of DEGs (up- and down-regulated) was significantly higher in HY than in MH (Figure 2A). These results suggest that more DEGs are involved in disease resistance and a large-scale response to pathogen infection in HY.

### 2.3. Functional Classification of DEGs after Psa Inoculation

**Figure 3:**
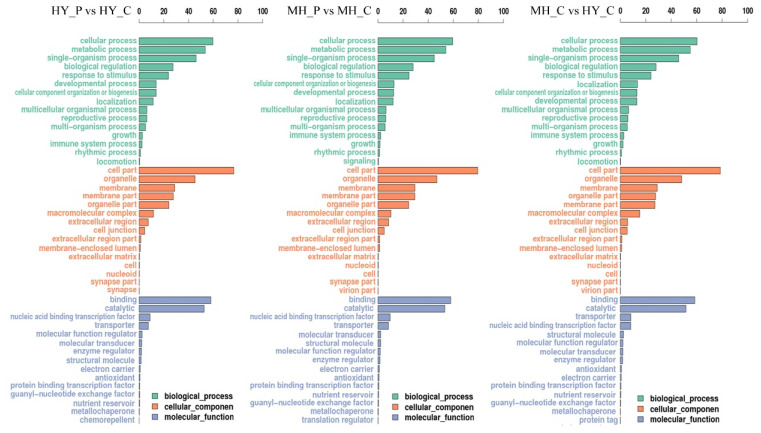
GO-enrichment analysis of the DEGs in comparisons between MH and HY after Psa infection.

GO analysis was performed to clarify the functions of DEGs in comparisons between the two varieties after Psa infection. GO can be divided into three categories: biological process, cellular component, and molecular function. In the category of biological process, GO, in terms of cellular process, metabolic process, and single-organism process, enriched the most DEGs (Figure 3). In the category of cellular component, the cell part, organelle, and membrane enriched the most DEGs. Binding and catalytic activity acquired the most DEGs in the category of molecular function. Moreover, a large number of DEGs in HY_P compared with HY_C were associated with the significantly enriched in process, as related to the response to organic substance, endogenous stimulus, and hormone. Interestingly, a lot of DEGs in comparison to MH_P and MH_C were significantly enriched in processes such as the single-organism biosynthetic process, carbohydrate metabolic process, cell-wall organization or biogenesis, and polysaccharide-metabolic process, which were involved in cell-wall defense.

### 2.4. Pathway-Enrichment Analysis of DEGs after Psa Inoculation

We compared the expression patterns between the Psa-treated samples and the control for two varieties, respectively. According to GO analysis, we focused on the pathways related to stimulus and hormones, the carbon metabolic process, and cell-wall organization or biogenesis, which were reported to be associated with plant-immunity processes [21,22]. There were also many DEGs participating in the biosynthesis of secondary metabolites, environmental adaptation, and carbohydrate metabolism. Among the most differentially expressed genes, we identified DEGs participating in plant hormone-signal transduction, plant–pathogen interaction, protein processing in endoplasmic reticulum, the cell cycle, and the peroxisome between HY_P and HY_C. We also identified DEGs participating in antigen processing and presentation, plant hormone-signal transduction, galactose metabolism, and the phagosome between MH_P and MH_C.

The metabolic process enriched the most abundant DEGs in the top 20 processes. Four processes, plant hormone signal transduction, plant–pathogen interaction, regulation of peroxisome, and phenylpropanoid biosynthesis, were directly related to the plant response to stress in HY. While the processes of plant hormone signal transduction, regulation of unsaturated fatty acids, and regulation of phagosome were related to the plant response to stress in MH (Table 2). KEGG analysis showed that the pathway of translation was the mostly influenced pathway and enriched the most abundant DEGs in both varieties. Other pathways influenced by Psa treatment included secondary metabolites and the metabolism of carbohydrates, amino acids, and lipids.

### 2.5. Analysis of Genes Related to Plant Innate Immune System

**Figure 4:**
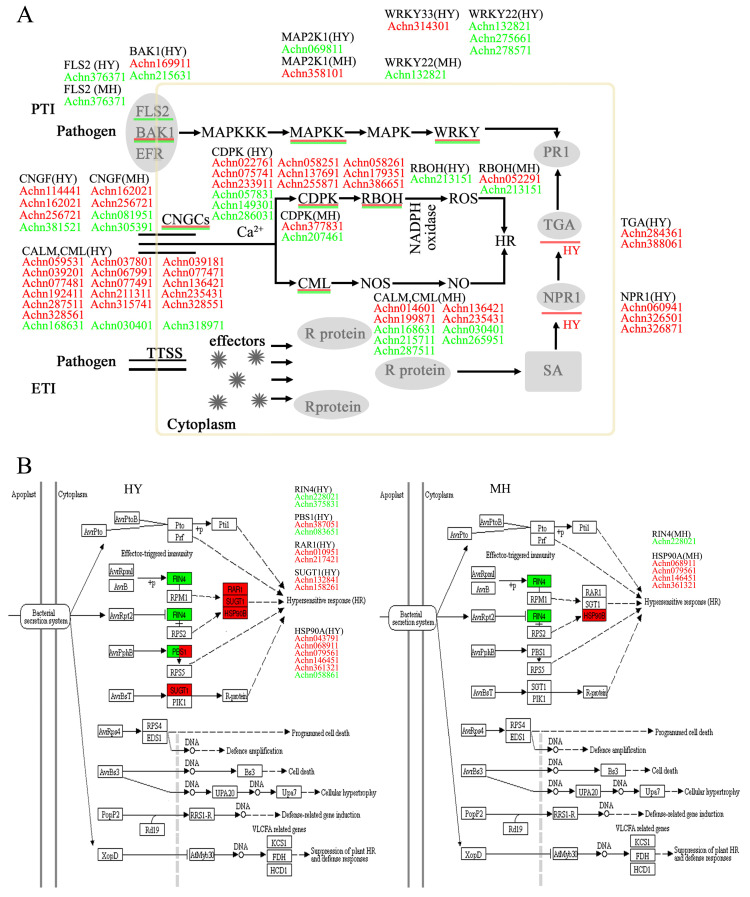
Expression patterns of the DEGs involved in PTI and ETI in response to Psa infection in MH and HY. (**A**) Schematic diagram of the plant’s innate immune-system response to Psa inoculation. (**B**) Expression patterns of the DEGs involved in ETI between MH and HY. Gene-expression level was measured using the FPKM method (expected number of fragments per kilobase of transcript sequence per millions of base pairs sequenced). The map shows the expression of genes in red (up-regulated expression) and green (down-regulated expression).

Plants have an immune system, which is divided into two main mechanisms, namely PTI and ETI [23,24]. In our study, 60 DEGs and 16 DEGs were involved in the plant innate immune system in HY and MH, respectively (Figure 4). Activation of FLS2 and EFR triggers the MAPK signaling pathway, which activates defense genes for antimicrobial compounds. In Arabidopsis, flg22 is recognized by FLS2, which subsequently activates the mitogen-activated protein kinase cascade and the downstream transcription factor WRKY [25,26]. WRKY transcription factors, which play a critical role in plant-defense response, have both positive and negative regulators [27]. One DEG encoding the WRKY33 gene (Achn314301) was much higher in HY after Psa infection. The expression of somatic embryogenesis receptor kinase genes (SERK4) (Achn169911, Achn215631) were also identified in response to Psa. The one (Achn169911) was up-regulated, while the other one (Achn215631) was down-regulated after Psa infection. Meanwhile, the MAP2K1 gene (Achn358101) and two glycerol kinase genes (Achn130621, Achn310081) were also identified and, obviously, up-regulated in MH after Psa infection. The PTI-related gene responses after Psa infection revealed their different regulation response between HY and MH against Psa in different ways for PTI.

Furthermore, pathogen effectors activate ETI, and result in plant-disease resistance and a hypersensitivity response. This response can inhibit the growth, reproduction, and expansion of pathogenic bacteria [28,29]. To clarify the role of ETI in defense response after Psa infection, we focused on the expression of AvrRpt2- and AvrPphB-related genes. Two disease resistance protein (RAR1) genes (Achn010951, Achn217421), two suppressor of G2 allele of SKP1 (SUGT1) genes (Achn132841, Achn158261) and five HSP90A genes (Achn043791, Achn068911, Achn079561, Achn146451, Achn361321) related to AvrRpt2 were found to be up-regulated in HY after Psa infection, while one HSP90A gene (Achn058861) was down-regulated. Two serine/threonine-protein kinase (PBS1) genes related to AvrPphB were also identified. One PBS1 gene (Achn387051) was up-regulated, and another one (Achn083651) was down-regulated, in response to Psa. In contrary, just four HSP90A genes (Achn068911, Achn079561, Achn146451, Achn361321) were up-regulated in MH after Psa infection. The expression pattern of the four HSP90A genes was the same for HY and MH against Psa. In addition, the DEGs of NPR1 and TGA were only up-regulated in HY after Psa infection, which were involved in the SA signaling pathway as regulatory roles of plant innate immunity. The patterns indicate that resistance genes were focused on the AvrRpt2-related pathway, and that the ETI reaction was much stronger in HY than in MH.

### 2.6. Regulation of Ca^2+^ Internal Flow and Reactive-Oxygen Species

The increase in the cytosolic Ca^2+^ concentration is also a regulator for production of reactive-oxygen species and localized programmed cell-death/hypersensitive response. The plant cyclic nucleotide gated channel (CNGF) is an important part of plant hypersensitivity. The channels can participate in the regulation of Ca^2+^ internal flow [30,31]. Six genes encoding the cyclic nucleotide gated channels were assessed in two varieties. Four of six genes were found to respond to Psa in HY and MH, respectively, and three genes were up-regulated in HY (Figure 4A). In particular, Achn162021 and Achn256721 were up-regulated in both varieties after Psa infection. More genes encoding these cyclic nucleotide gated channels were up-regulated in HY after Psa infection. Meanwhile, Ca^2+^ when combined with a calcium-binding protein (CML) produces NO, which further promotes plant-hypersensitivity responses or autoimmune reactions [32]. We found 23 homologs of CML that were responsive to Psa in HY and MH. Notably, 16 of 19 genes were found to be up-regulated in HY, while 5 of 9 genes were down-regulated in MH. Two genes (Achn136421, Achn235431) were up-regulated higher in HY than in MH, and another two genes (Achn168631, Achn030401) were down-regulated in both varieties. Interestingly, Achn287511 was three-fold up-regulated in HY, while three-fold down-regulated in MH. This showed that more homologs of CML were responsive to Psa in HY than in MH, and the expression of most homologs was increased in HY after Psa infection.

Furthermore, Ca^2+^ can also activate calcium-dependent protein kinases (CDPKs), and then phosphorylate downstream targets proteins and activates respiratory burst oxidase homolog (RBOH) activity [33,34]. RBOH, also known as NADPH oxidase, produces reactive-oxygen species and plays a critical role in plant responses to abiotic and biotic stresses [35,36]. In our study, the changes in the expression of CDPK- and ROBH-like genes were observed in both cultivars in response to Psa infection. Twelve genes of the homologs of CDPK were found response to Psa in HY, and nine genes were up-regulated, while three genes were down-regulated. In comparison, just one gene (Achn377831) was up-regulated and one gene (Achn207461) was down-regulated in MH. One gene encoding ROBH (Achn213151) was down-regulated in response to Psa in both varieties. In particular, one ROBH-like gene (Achn052291) was up-regulated in MH (Figure 4). The results found CDPK- and ROBH-like genes were more responsive to Psa in HY than in MH. These revealed that Ca^2+^ participated in signal transduction in response of Psa infection in kiwifruit, especially in HY. The role of reactive oxygen species was stronger in HY than in MH, which was also supported by the browning phenomenon observed on the HY shoots.

### 2.7. Analysis of DEGs Involved in Secondary Metabolism

Multiple pathways involved in the metabolism of secondary metabolites were enriched, including those related to the metabolism of the three main kinds of secondary metabolites: terpenes, phenols, and alkaloids (Tables S3 and S4). Terpenes are the richest natural production and all of the monoterpenoid, diterpenoid, triterpenoid, and sesquiterpenoid (carotenoid) and terpenoid backbone biosynthesis pathways were regulated by Psa infection. Expression of as many as 21 genes in the phenylpropanoid biosynthesis pathway, which is the key process in the biosynthesis of phenols, was up-regulated in HY. Gene encoding the key enzyme 4-coumarate-CoA ligase (4CL) in phenol biosynthesis was found to be regulated. Meanwhile, just 18 DEGs in phenylpropanoid biosynthesis pathway was found, and the gene encoding the key enzyme cinnamoyl-CoA reductase (CCR) was found to be up regulated in MH. Metabolism of the three main intermediate products phenylalanine, tyrosine, and tryptophan of phenol biosynthesis was also influenced. Two pathways in alkaloids biosynthesis were changed, but the number of genes regulated only accounted for a small part of all the genes annotated.

**Figure 5:**
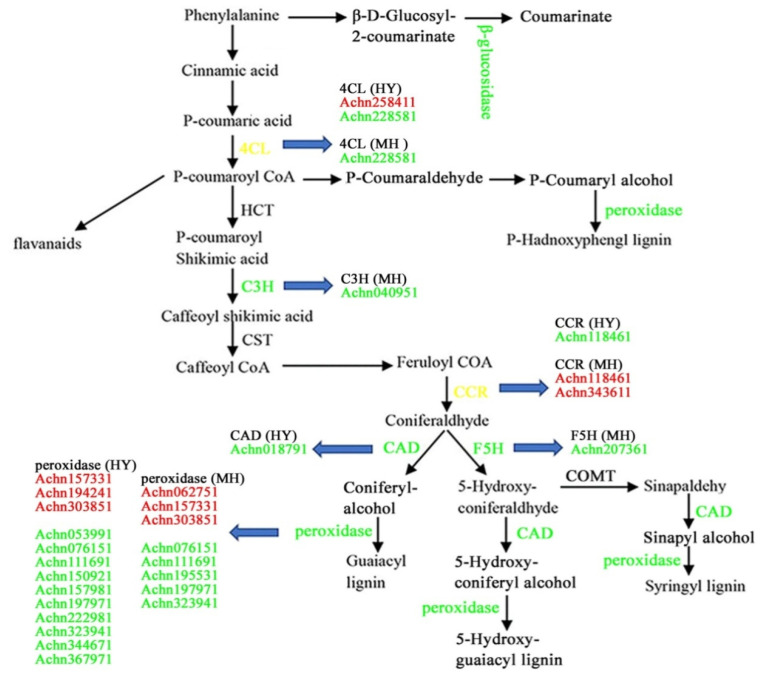
Expression patterns of the DEGs involved in the biosynthesis of phenylpropanoid, in response to Psa infection in MH and HY. Gene-expression level was measured using the FPKM method (expected number of fragments per kilobase of transcript sequence per millions of base pairs sequenced). The map shows the expression of genes in red (up-regulated expression) and green (down-regulated expression). The expressed genes represented are 4-hydroxycinnamoly CoA ligase (4CL), coumaroylquinate 3-monooxygenase (C3H), cinnamoyl-CoA reductase (CCR), cinnamyl alcohol dehydrogenase (CAD), ferulate-5-hydroxylase (F5H), and peroxidase.

Plant phenols are secondary metabolites, working as signal compounds, pigments, internal physiological regulators, or chemical messengers and functioning in the resistance mechanism of plants against pathogens [37]. Most phenols biogenetically arise from the phenylpropanoid pathway, where some key genes play a key role in phenols production. In our study, a total of 22 DEGs were involved in lignin biosynthesis, including 4-coumarate-CoA ligase (4CL) (two genes), coumaroylquinate (coumaroylshikimate) 3-monooxygenase (C3H) (one gene), cinnamoyl-CoA reductase (CCR) (two genes), ferulate-5-hydroxylase (F5H) (one gene), cinnamyl-alcohol dehydrogenase (CAD) (one gene), and peroxidase (fifteen genes); most of the genes were down-regulated in both varieties after Psa infection (Figure 5). Notably, the expression levels of the 4CL-like gene (Achn258411) and three homologs of peroxidase gene (Achn157331, Achn303851, Achn194241) were significantly up-regulated in HY. Meanwhile, two homologs of the CCR gene (Achn118461, Achn343611) and three peroxidase genes (Achn157331, Achn303851, Achn062751) were also up-regulated in MH. The homolog of the C3H gene (Achn040951) and F5H gene (Achn207361) were just observed and down-regulated in MH in response to Psa infection. Interestingly, the CCR homolog gene (Achn118461) was down-regulated in HY after Psa infection, but up-regulated in MH, which displayed a large difference between HY and MH. In particular, previous studies have reported that glutathione S-transferase (GST) played an important role in flavonoid biosynthesis [38,39]. We found thirteen GST-like genes that were significantly up-regulated in HY, compared to five genes that were up-regulated in MH after Psa infection (Tables S1 and S2).

### 2.8. Verification of Gene Expression by q-PCR

To confirm the validity of the RNA-Seq, five DEGs were randomly chosen for qRT-PCR (Figure S2). All of the selected genes exhibited similar expression patterns to those from the RNA-seq data, indicating that the results of RNA sequencing were credible.

## 3. Discussion

Plants lack animal-like, adaptive immunity mechanisms, and, therefore, have evolved a specific system with multiple layers against various invasion. Besides these defense mechanisms, plants also have evolved a specific immune system, namely PTI and ETI, to defend themselves against various pathogens [23,24]. Our study found the WRKY33 gene and SERK4 genes were varied in HY against Psa, and the MAP2K1 gene and glycerol kinase genes were up-regulated in MH after Psa infection. This difference revealed that the HY and MH respond to PSA in different ways for PTI. The results were also revealed in relevant studies on the model plants, *Arabidopsis* [27].

A series of effectors could be involved by pathogens, and then interact with resistance proteins to elicit the second response named ETI. ETI activation leads to plant-disease resistance and hypersensitivity. This hypersensitivity response can inhibit the growth, reproduction, and expansion of pathogenic bacteria [28,29]. Tomato studies reported that Pto as a resistant protein against *Pseudomonas syringae* pv. *tomato* (Pst), can recognize the pathogenic bacteria effector proteins AvrPtoB and AvrPto. Then, it triggers the downstream defense reaction, and eventually produces a hypersensitivity reaction at the infection site to prevent the colonization of pathogenic bacteria. Pto-interaction protein (Pti) genes, *Pti4* and *Pti5*, can further improve tomato resistance to Pst [40,41]. The expression of AvrRpt2- and AvrPphB-related genes was focused, to clarify the role of ETI in the defense response after Psa infection, in our studies. RAR1 genes, SUGT1 genes, AvrRpt2- and AvrPphB-related genes such as HSP90A were found to act against Psa. Most of them were varied in HY after Psa infection, which indicated that the ETI reaction was stronger to act against Psa in HY than in MH.

In general, secondary metabolites, such as lignin, play a critical role in the struggle between plants and pathogens. Studies have shown that when plants are infected by pathogenic bacteria, lignin is synthesized in large quantities at the infection site, strengthening the lignification of plant-cell walls and resisting further infection by the pathogenic bacteria. The phenylpropanoid pathway mainly synthesizes lignin and flavonoids [42,43]. When *Gossypium hirsutum* was inoculated with pathogenic bacteria, a large amount of lignin was synthesized in the stem, which improved its resistance to verticillium wilt [44]. Our study showed a total of 22 DEGs involved in lignin biosynthesis were different between HY and MH after Psa infection, which also revealed a different resistance in response to Psa.

Plant pathogens are divided into three categories: biotrophs, hemibiotrophs, and necrotrophs, according to their lifestyles. It is clear that the SA signaling pathway mediates against biotrophic and hemibiotrophic pathogens to activate defense responses [45]. SA is a critical regulator in plant–pathogen interactions, and it can induce plant hypersensitive responses and systematic acquired resistance [46,47]. In our study, all of the DEGs involved in the SA signaling pathway (NPR1 and TGA) were only up-regulated in HY after Psa infection (Figure 4). Importantly, the expression of the TGA genes (Achn284361, Achn388061), an important regulator acting downstream of the SA signaling pathway, interacting with NPR1 to positively regulate the expression of PR1 and the plant’s disease-resistance response [48,49], were just up-regulated to 17-fold in HY. In addition, we also found that the NPR1-like genes (Achn060941, Achn326501, Achn326871) were about three-fold higher after Psa infection. (Figure S1 and Table S1) The results obviously show an induced SA signaling pathway in HY after Psa infection. In contrast, the jasmonic acid (JA)/ethylene (ET) signaling pathway is mainly resistant to necrotrophic pathogens, and the SA and JA/ET signaling pathways are antagonistic to each other [50]. The genes involved in the JA/ET signaling pathway were also identified. The JA signaling component JAR1, JAZ, and ET signaling components EIN3 and ERF1 were, obviously, found to respond to Psa in HY and MH, respectively. Notably, two ERF1-related genes (Achn352361, Achn359661) were down-regulated in HY but one (Achn334501) was up-regulated in MH after Psa infection. More interestingly a JAZ homolog gene (Achn360591) and a transcription factor MYC2 homolog gene (Achn281761) involved in JA signaling pathway were dramatically down-regulated in HY, which might offset the up-regulation of the JAR1 (Achn275921) in upstream. Similarly, three JAZ homolog genes (Achn061431, Achn240451, Achn360591) were also down-regulated in MH after Psa infection. Furthermore, our experiment found a transcription-factor WRKY70-like gene (Achn258601) that was up-regulated in HY but down-regulated in MH after Psa infection (Tables S1 and S2). WRKY70, as a common component, is an activator in the SA signaling pathway but an inhibitor in the JA signaling pathway [51]. In addition, an antagonistic interaction was also reported between the SA- and abscisic acid (ABA)-mediated signaling pathways in *Arabidopsis* [52]. We found that all of the *PP2C* and *ABF* genes related to the ABA signaling pathway were up-regulated in both shoots after Psa infection, which suggested that ABA might have positive regulatory effects during Psa infection. However, just four *PYR/PYL* genes and one *SNRK2* gene were down-regulated in HY shoots, and two *PYR/PYL* genes down-regulated in MH (Figure S1). These results indicate that the SA signaling pathway, but not the JA/ET or ABA signaling pathways, played a pivotal role in the defense response of HY shoots after Psa inoculation, to improve kiwifruit resistance.

Auxin was also involved in the plant disease-resistance response. The auxin level of *Arabidopsis*, lacking a functional RPS2 gene (rps2 mutant), is increased after infection with *Pseudomonas syringae* pv. *tomato* strain DC3000 (PstDC3000); the resistance of the rps2 mutant is also decreased [53]. The resistance of the auxin-insensitive axr2-1 *Arabidopsis* mutation is improved during PstDC3000 infection, suggesting that the auxin signaling pathway is harmful to the *Arabidopsis* immune response to PstDC3000 infection [54]. In our study, most of the genes such as *AUX1*, *IAA, ARF, GH3*, and *SAUR* involved in the AUX signaling pathway were down-regulated in the HY shoots after Psa infection. However, the SAUR homolog gene (Achn321001) was up-regulated in the MH shoots after Psa infection. All results suggested that AUX might have negative regulatory effects during Psa infection (Figure S1).

Cytokinin regulated the SA signaling pathway to promote the resistance of *Arabidopsis* to PstDC3000 [55]. It was also reported that brassinosteroids (BRs) could enhance wild-type tobacco resistance to the viral pathogen tobacco mosaic virus and the bacterial pathogen *Pseudomonas syringae* pv. tabaci [56]. Our study found the genes involved in the CTK signaling pathway, for example *AHK* genes and *ARR* genes, were up-regulated in HY. In contrast, the genes encoding the BR signaling pathway were down-regulated in HY and MH, as a result of the kiwifruit plant’s response to Psa (Figure S1).

Together, these results indicate that the SA and CTK signaling pathways played a positive role in HY against Psa. The DEGs involved in the SA and CTK signaling pathways were up-regulated in the HY shoots, whereas the most of genes involved in the AUX and BR signaling pathways were down-regulated in the HY shoots. The genes involved in phytohormone signaling pathways were less in MH than in HY, which might indicate that phytohormone signaling pathways are less participated in MH against Psa. The different expression patterns of multiple phytohormone signaling pathways indicated that the phytohormone signaling pathways are not independent of each other but that there is crosstalk amongst them.

## 4. Materials and Methods

### 4.1. Plant Materials and Psa Inoculation

*Actinidia eriantha* Bentham cv youxi (MH) were grown in the *Actinidia* germplasm resources repository of the Zhengzhou Fruit Research Institute, Chinese Academy of Agricultural Sciences, located in Zhengzhou, Henan province, China. *Actinidia chinensis* Planchon cv HY (HY) were grown in the *Actinidia* germplasm resources base of Kiwifruit Breeding and Utilization Key Laboratory of Sichuan Province, Sichuan Provincial Academy of Natural Resource Sciences, located in Shifang, Sichuan province, China. MH has been proven to be resistant to Psa, while HY is susceptible to it. One-year-old shoots, approximately 0.8 cm in diameter, were collected from the vines in April. The pathogen Psa was provided by the Kiwifruit Breeding and Utilization Key Laboratory of Sichuan Province. Psa was cultured on beef peptone medium for 24 h at 20 °C. The microbial concentration of Psa was diluted to 10^8^ colony-forming units (cfu)/ml prior to inoculation. For inoculation, the detached shoots were surface sterilized with chlorine and then cut into 10 cm shoots, and the ends of the shoots were dipped in candle wax to reduce dehydration. A wound was made with a file about 1–1.5 cm from each end of the shoot, and Psa was added to the wound with a pipette. Control shoots were treated with sterile water. The inoculated and control shoots were placed in trays, which were placed in an artificial climate incubator at 20 °C and 80% relative humidity for 12 h day/night cycles. Every three samples were taken from one shoot, and five plant shoots were taken as biological independent duplicates.

Shoot samples were taken from the inoculated and mock-inoculated segments 0.5–1 cm away from the wound point at 0 and 72 h after inoculation (hai). The samples were immediately placed in liquid nitrogen and stored at −80 °C for RNA extraction and further analysis. The samples were collected with three biological replicates.

### 4.2. Total RNA Extraction, cDNA Library Construction, and Sequencing

Total RNA was extracted using the RNA prep Pure Plant Kit (Polysaccharides & Polyphenolics-rich, Tiangen, Beijing, China), in accordance with the protocol of the manufacturer. RNA concentration was measured using the Qubit RNA Assay Kit in a Qubit 2.0 Flurometer (Life Technologies, Carlsbad, CA, USA), and RNA integrity was assessed using the RNA Nano 6000 Assay Kit of the Bioanalyzer 2100 system (Agilent Technologies, Santa Clara, CA, USA). Following RNA quantification and qualification, mRNA was purified from total RNA using poly-T oligo-attached magnetic beads, and the mRNA was then broken into fragments using a fragmentation buffer. First-strand cDNA was synthesized using random hexamer primers, and second-strand cDNA synthesis was subsequently performed using buffer, dNTPs, DNA Polymerase I, and RNase H. The library fragments were purified with AMPure XP beads (Beckman Coulter, Beverly, MA, USA), and USER enzyme was used with size-selected, adaptor-ligated cDNA before PCR. PCR was performed to enrich the purified cDNA libraries. Finally, the library preparations were sequenced on an Illumina HiSeq platform.

### 4.3. Sequencing Read Mapping and Identification of DEGs

Raw reads in FASTQ format were generated by base calling. Clean reads were obtained by removing reads with adapters, reads containing more than 10% ploy-N (where N refers to unknown bases), and low-quality reads. All subsequent analyses were based on the clean data, which were aligned to the reference genome using TopHat v2.0.12 [57]. Gene-expression levels were calculated using the FPKM method (expected number of fragments per kilobase of transcript sequence per millions of base pairs sequenced) using HTSeq v0.6.1 [58]. DEGs were analyzed using the DESeq R package (1.18.0) [59]. Essentially, differential-expression analysis was performed using DESeq2/DEGseq/EdgeR with Q value ≤ 0.05; DEGs with |log2FC| > 1 and Q value ≤ 0.05 (DESeq2 or EdgeR)/Q value ≤ 0.001 (DEGseq) were considered to be significantly different expressed genes [60].

### 4.4. Gene Ontology and KEGG Pathway Analysis

BLASTx program (version 2.2) was used to annotate the unigenes, with an E-value threshold of 10^−5^ to NCBI non-redundant protein (Nr) database (http://www.ncbi.nlm.nih.gov, accessed on 14 June 2022), the Swiss-Prot protein database (http://www.expasy.ch/sprot, accessed on 14 June 2022), and the COG/KOG database (http://www.ncbi.nlm.nih.gov/COG, accessed on 14 June 2022). The best alignment results were for protein-functional annotations. Kyoto Encyclopedia of Genes and Genomes (KEGG) annotations were obtained from http://www.genome.jp/kegg, accessed on 14 June 2022.

Gene ontology (GO)-enrichment analysis of DEGs was executed using the R package [61]. Gene-ontology terms with corrected *p* < 0.05 were considered significantly enriched in DEGs. KEGG pathway-enrichment analysis was done to identify significantly enriched metabolic pathways or signal transduction pathways in DEGs, compared with the whole genome background.

### 4.5. Quantitative Real-Time PCR Analysis

To test the expression results from the RNA-seq analysis, we determined the expression levels of the selected genes by the q-PCR method. Primers were designed using Primer5 software (Table S5), and 2×SYBR Green I RT-PCR Master Mix (Roche) was used as a fluorescent reporter. PCR was performed with condition of pre-incubation for 5 min at 95 °C, followed by 40 cycles of 95 °C for 20 s, 60 °C for 20 s, and 72 °C for 20 s. The relative gene expression was calculated using the 2-ΔΔc(t) method.

## Figures and Tables

**Table 1:** Sequencing statistics of the transcriptome samples.

Samples	Raw Reads	Clean Reads	Mapped Reads	Uniquely Mapped	Uniquely Mapped Rate
HY_C_1	53173060	47659800	42445216	41754675	87.61%
HY_C_2	50688944	44697010	39824177	38533070	86.21%
HY_C_3	50321846	45046286	39975928	39282108	87.20%
HY_P_1	52583390	47199212	40516165	39783757	84.29%
HY_P_2	47920524	42513064	36786553	35602846	83.75%
HY_P_3	52116128	46209178	41318821	40630090	87.93%
MH_C_1	47134410	41928396	16033373	14770334	35.23%
MH_C_2	51174634	45465026	17584600	16821959	37.00%
MH_C_3	53840934	47798112	18719524	17294295	36.18%
MH_P_1	51884426	45765796	17705514	17016783	37.18%
MH_P_2	50151192	44200726	17146800	15883761	35.94%
MH_P_3	53392744	47376418	18078332	16653103	35.15%

HY_C, HY_P, MH_C, and MH_P represent the control sample of HY, the HY sample inoculated with Psa, the control sample of MH, and the MH sample inoculated with Psa, respectively; 1, 2, and 3 represent the relevant number of the three biological replicates.

**Table 2:** The top 20 enriched pathways involved in secondary metabolism.

Description	DEGs of HY			Description	DEGs of MH		
	Up	Down	All		Up	Down	All
Metabolic pathways	153	232	385	Metabolic pathways	116	178	294
Biosynthesis of secondary metabolites	78	141	219	Biosynthesis of secondary metabolites	11	96	107
Plant hormone signal transduction	36	46	82	Starch and sucrose metabolism	30	39	69
Microbial metabolism in diverse environments	41	38	79	Antigen processing and presentation	58	7	65
Plant–pathogen interaction	40	20	60	Microbial metabolism in diverse environments	5	33	38
Biosynthesis of amino acids	15	34	49	Plant hormone-signal transduction	9	27	36
Starch and sucrose metabolism	13	33	46	Amino sugar and nucleotide sugar metabolism	9	24	33
Protein processing in endoplasmic reticulum	32	10	42	Biosynthesis of unsaturated fatty acids	26	6	32
Amino sugar and nucleotide sugar metabolism	6	31	37	Phagosome	7	19	26
Glycolysis/Gluconeogenesis	14	19	33	Biosynthesis of amino acids	1	24	25
Cell cycle	5	26	31	Galactose metabolism	16	8	24
Peroxisome	20	10	30	Purine metabolism	4	20	24
Pentose and glucuronate interconversions	6	24	30	Pentose and glucuronate interconversions	8	15	23
Purine metabolism	6	23	29	Glycolysis/gluconeogenesis	5	18	23
Cysteine and methionine metabolism	7	21	28	Protein processing in endoplasmic reticulum	2	20	22
Pyrimidine metabolism	9	18	27	Photosynthesis	7	11	18
Phenylalanine metabolism	9	15	24	Gap junction	11	6	17
Glutathione metabolism	18	5	23	Estrogen-signaling pathway	15	1	16
Epstein-Barr virus infection	12	10	22	Cell cycle	2	14	16
Phenylpropanoid biosynthesis	6	15	21	Plant–pathogen interaction	1	15	16

## Supplementary Materials

The following supporting information can be downloaded at: https://www.mdpi.com/article/10.3390/ijms23147643/s1.
