# Supplementary material for: Transcriptional Analysis on Resistant and Susceptible Kiwifruit Genotypes Activating Different Plant-Immunity Processes against Pseudomonas syringae pv. actinidiae
Source: Int J Mol Sci. 2022 Jul 11;23(14):7643. doi: 10.3390/ijms23147643 (PMC9322148; doi:10.3390/ijms23147643)
Supplement: Supplementary file 1 [file ijms-23-07643-s001.zip › Figure S2 q-PCR validation.pdf]

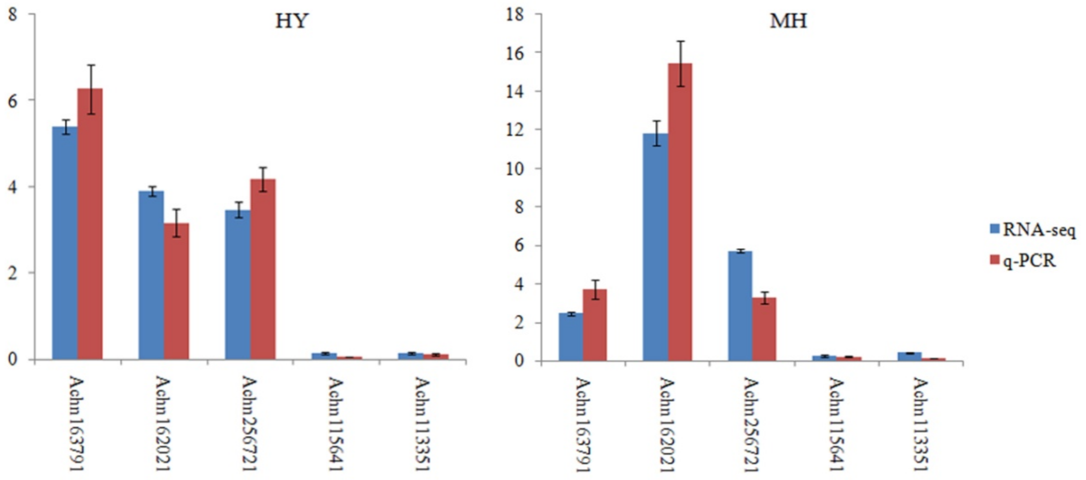

Figure S2. Expression levels of the selected genes from RNA sequencing data and q-PCR in MH and HY. Expression values were displayed by the fold of Psa inoculation to Control.
