# Supplementary material for: Transcriptional Analysis on Resistant and Susceptible Kiwifruit Genotypes Activating Different Plant-Immunity Processes against Pseudomonas syringae pv. actinidiae
Source: Int J Mol Sci. 2022 Jul 11;23(14):7643. doi: 10.3390/ijms23147643 (PMC9322148; doi:10.3390/ijms23147643)
Supplement: Supplementary file 1 [file Figure S1 Plant hormone signal HY MH.pdf › Figure S1 Plant hormone signal HY MH.pdf]

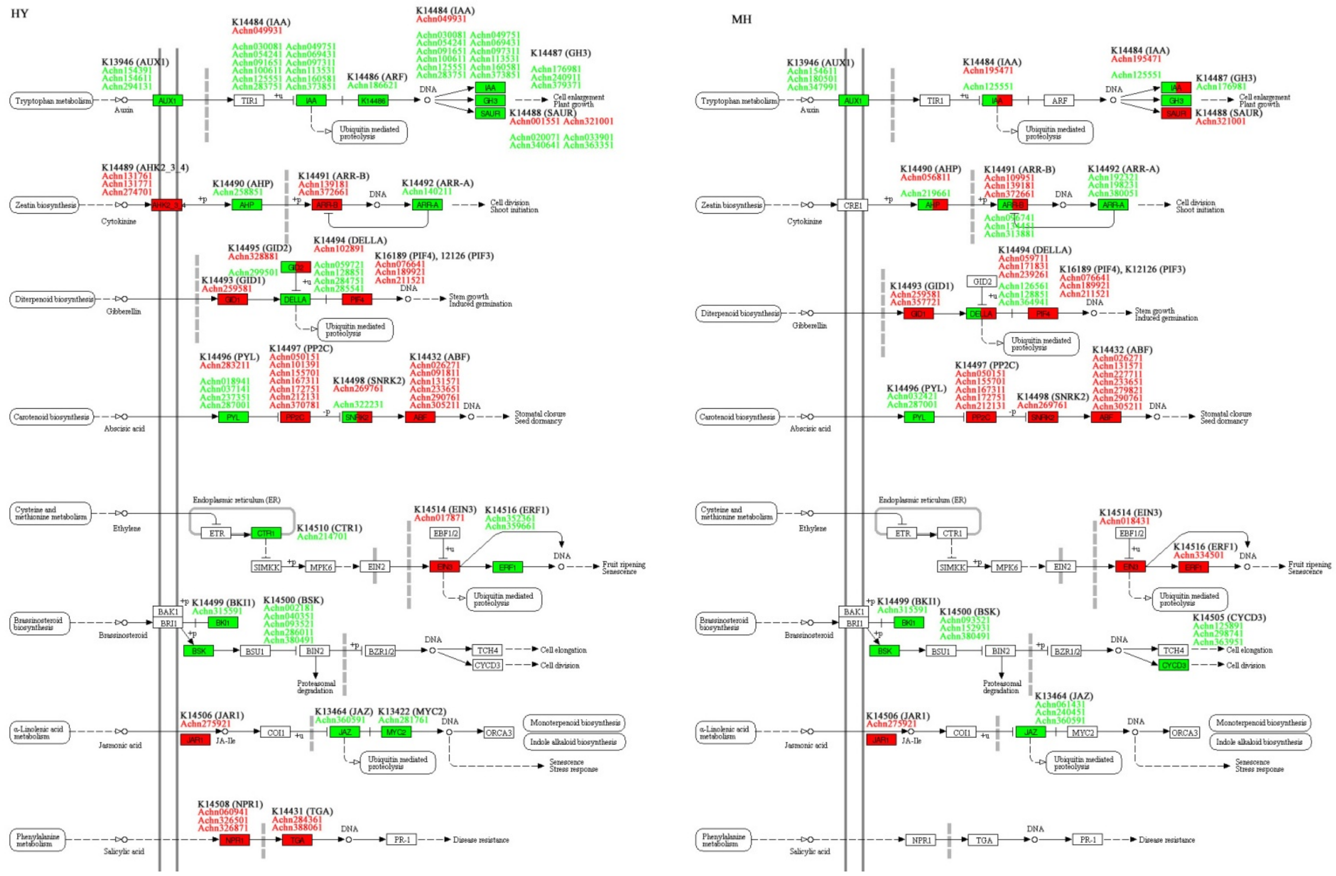

Figure S1. Expression patterns of the DEGs involved in phytohormone signaling pathways in response to Psa infection in MH and HY.

Gene expression level was measured using the FPKM method (expected number of fragments per kilobase of transcript sequence per millions of base pairs sequenced). The map shows the expression of genes with red (up-regulated expression) and green (down-regulated expression).
